# Susceptibility of Type I Interferon Receptor Knock-Out Mice to Heartland Bandavirus (HRTV) Infection and Efficacy of Favipiravir and Ribavirin in the Treatment of the Mice Infected with HRTV

**DOI:** 10.3390/v14081668

**Published:** 2022-07-28

**Authors:** Hikaru Fujii, Hideki Tani, Kazutaka Egawa, Satoshi Taniguchi, Tomoki Yoshikawa, Shuetsu Fukushi, Souichi Yamada, Shizuko Harada, Takeshi Kurosu, Masayuki Shimojima, Takahiro Maeki, Chang-Kweng Lim, Mutsuyo Takayama-Ito, Takashi Komeno, Nozomi Nakajima, Yousuke Furuta, Akihiko Uda, Shigeru Morikawa, Masayuki Saijo

**Affiliations:** 1 The Faculty of Veterinary Medicine, Okayama University of Science, 1-3 Ikoino-oka, Imabari 794-8555, Japan; s-morikawa@ous.ac.jp; 2 Department of Virology I, National Institute of Infectious Diseases, 1-23-1 Toyama, Shinjuku-ku, Tokyo 162-8640, Japan; toyamaeiken3@juno.ocn.ne.jp (H.T.); egawa@niid.go.jp (K.E.); rei-tani@niid.go.jp (S.T.); ytomoki@niid.go.jp (T.Y.); fukushi@niid.go.jp (S.F.); syamada@niid.go.jp (S.Y.); shizuko@niid.go.jp (S.H.); kurosu@niid.go.jp (T.K.); shimoji-@niid.go.jp (M.S.); tomaeki@niid.go.jp (T.M.); ck@niid.go.jp (C.-K.L.); mutsuito@niid.go.jp (M.T.-I.); masayuki.saijo@doc.city.sapporo.jp (M.S.); 3 Department of Virology, Toyama Institute of Health, 17-1 Nakataikouyama, Imizu-shi 939-0363, Japan; 4 FUJIFILM Toyama Chemical Co., Ltd., 2-4-1 Shimookui, Toyama City 930-8508, Japan; takashi.komeno@fujifilm.com (T.K.); nozomi.nakajima@fujifilm.com (N.N.); yousuke.furuta@fujifilm.com (Y.F.); 5 Department of Veterinary Science, National Institute of Infectious Diseases, 1-23-1 Toyama, Shinjuku-ku, Tokyo 162-8640, Japan; auda@niid.go.jp; 6 Sapporo City Health & Welfare Bureau, Public Health Office, WEST 19, Chuo-ku West 19, Sapporo 060-0042, Japan

**Keywords:** Heartland bandavirus, animal model, favipiravir, ribavirin

## Abstract

Heartland bandavirus (HRTV) is an emerging tick-borne virus that is distributed in the United States and that causes febrile illness with thrombocytopenia and leukocytopenia. It is genetically close to Dabie bandavirus, which is well known as severe fever with thrombocytopenia syndrome (SFTS) virus (SFTSV). The mortality rate of human HRTV infection is approximately 10%; however, neither approved anti-HRTV agents nor vaccines exist. An appropriate animal model should be developed to evaluate the efficacy of antiviral agents and vaccines against HRTV. The susceptibility of IFNAR^−/−^ mice with HRTV infection was evaluated using subcutaneous, intraperitoneal, and retro-orbital inoculation routes. IFNAR^−/−^ mice intraperitoneally infected with HRTV showed the most severe clinical signs, and the 50% lethal dose was 3.2 × 10^6^ TCID_50_. Furthermore, to evaluate the utility of a novel lethal IFNAR^−/−^ mice model, IFNAR^−/−^ mice were orally administered favipiravir, ribavirin, or a solvent for 5 days immediately after a lethal dose of HRTV inoculation. The survival rates of the favipiravir-, ribavirin-, and solvent-administered mice were 100, 33, and 0%, respectively. The changes in bodyweights and HRTV RNA loads in the blood of favipiravir-treated IFNAR^−/−^ mice were the lowest among the three groups, which suggests that favipiravir is a promising drug candidate for the treatment of patients with HRTV infection.

## 1. Introduction

Heartland bandavirus (HRTV) is a tick-borne virus that belongs to the genus *Bandavirus,* within the family *Phenuiviridae,* of the order *Bunyavirales*. HRTV is closely related to Dabie bandavirus, which is also known as severe fever with thrombocytopenia syndrome (SFTS) virus (SFTSV) [1]. SFTSV is also a tick-borne virus, which was first reported in China in 2011 [2]. More than 7000 cases of patients with SFTS have been reported in East Asia, including China, Korea, Japan, Taiwan, Vietnam, and Myanmar [2,3,4,5,6,7,8,9], while just more than 50 cases of human HRTV infection have been reported so far, which have only been reported in the United States [10,11,12]. However, as it was estimated that the prevalence of HRTV infection in blood-donor samples from northwestern Missouri in the United States was 0.9% [13], there might be a potential for undetected HRTV disease in this population.

The clinical symptoms of SFTS are fever, thrombocytopenia, leukocytopenia, and multiorgan dysfunction with a high mortality rate [2]. The clinical symptoms of HRTV infection are similar to those of SFTS [14,15]. The laboratory findings of SFTS include leukocytopenia and thrombocytopenia in the total blood-cell counts, and a mild-to-moderate elevation of liver transaminases was also found in patients infected with HRTV [11,12,15,16]. As both SFTS and HRTV infection show acute symptoms and high mortality rates, as described above, effective treatments are required. However, there are no approved antiviral drugs or vaccines against these infectious diseases.

Favipiravir is a purine nucleic acid analog that acts as a viral RNA polymerase inhibitor [17]. Ribavirin is a guanosine analog with a broad spectrum of antiviral activity [18]. Thus, both antiviral agents are supposed to inhibit viral RNA replication. In fact, both favipiravir and ribavirin were shown to be effective against SFTSV and HRTV infection in vitro [19,20]. The 90% inhibitory concentrations (IC_90_) for favipiravir and ribavirin against SFTSV infection in Vero cells were 22 μM and 117 μM, respectively [21]. The 90% effective concentrations (EC_90_) of favipiravir and ribavirin against HRTV infection in Vero E6 cells were 2.8 μg/mL ± 0.3 (17.5 μM) and 18.7 μg/mL ± 1.9 (76.6 μM), respectively [20]. The efficacy of favipiravir and ribavirin against SFTSV infection was also shown in vivo [21,22,23], while only favipiravir has been shown to be effective against HRTV infection in vivo [20].

Appropriate animal models are required to evaluate the efficacy of antiviral drugs and vaccines. Type I interferon receptor knock-out (IFNAR^−/−^) mice are commonly used to evaluate the efficacy of potential drugs and vaccines against SFTSV infection, as they are highly permissive to SFTSV infection and they mimic some SFTS signs in severe cases of human infection [21,23,24,25,26]. The susceptibilities of various animals to HRTV infection were examined in previous studies [20,27]. AG129 mice, which are deficient in both type I and type II interferon (IFN) signaling, are highly susceptible to HRTV infection [27]. HRTV infection induced a significant bodyweight decrease in IFNAR^−/−^ mice compared with the placebo group, but it was not lethal [20]. However, it was only investigated in weaning mice via intraperitoneal infection [20].

In this study, the susceptibilities of adult IFNAR^−/−^ mice to HRTV infection were compared on the subcutaneous, intraperitoneal, and retro-orbital inoculation routes, and a novel lethal HRTV-infection mouse model was established. Furthermore, the efficacies of antiviral agents such as favipiravir and ribavirin was evaluated using the IFNAR^−/−^ mouse model of HRTV infection developed in this study.

## 2. Materials and Methods

### 2.1. Cells and Viruses

Vero cells were cultured in Dulbecco’s modified Eagle’s essential medium (DMEM) (FUJIFILM Wako Chemicals, Osaka, Japan), supplemented with 5% fetal bovine serum (FBS) (Thermo Fisher Scientific K.K., Yokohama, Japan), 100 U/mL of penicillin, and 100 µg/mL of streptomycin (Thermo Fisher Scientific K.K.) (DMEM-5FBS).

The HRTV strain MO-4, kindly provided by Dr. Robert Tesh, the Department of Pathology and Microbiology and Immunology, the University of Texas Medical Branch, was passaged four times in Vero cells, was named HRTV strain MO-4-NIID [28], and was propagated and used in this study. HRTV strain MO-4-NIID was propagated and titrated using Vero cells cultured in DMEM, supplemented with 2% FBS, 100 U/mL of penicillin, and 100 µg/mL of streptomycin (DMEM-2FBS). All work involving the manipulation of infectious HRTV was performed in biosafety level-3 containment laboratories at the National Institute of Infectious Diseases (NIID), in accordance with the institutional biosafety operating procedures.

### 2.2. Animals

IFNAR^−/−^ mice, derived from C57BL/6J mice, were produced as described previously [21] and were maintained in an environmentally controlled specific-pathogen-free animal facility at the NIID. Male and female IFNAR^−/−^ mice, 7- to 11-weeks-old, were used in this study. All animal experiments were performed with the approval (No. 117082 and No. 117146) of the Animal Care and Use Committee of the NIID, in accordance with the approved guidelines.

### 2.3. Evaluation of Susceptibility of IFNAR^−/−^ Mice to HRTV Infection

IFNAR^−/−^ mice were anesthetized with midazolam, medetomidine, and a butorphanol tartrate combination, and were inoculated subcutaneously, intraperitoneally, or retro-orbitally with 1 × 10^7^ 50% tissue-culture-infective doses (TCID_50_) of HRTV, at a volume of 200 μL per mouse in each group, consisting of 3 mice, to examine the HRTV disease progression in terms of severity and outcome. Three IFNAR^−/−^ mice in each group were anesthetized with midazolam, medetomidine, and a butorphanol tartrate combination, and were inoculated intraperitoneally with 1 × 10^4^, 1 × 10^5^, or 1 × 10^6^ TCID_50_ of HRTV at a volume of 200 μL per mouse, whereas two mice were inoculated with 1×10^7^ TCID_50_ of HRTV at a volume of 200 μL per mouse to determine the LD_50_ of HRTV. Each mouse was monitored daily for the development of clinical signs, including bodyweight, and blood samples were obtained by tail-vein puncture at days 2, 4, 7, 11, and 14 postinfection to measure the viral RNA levels.

### 2.4. Evaluation of Antiviral Efficacy against HRTV Using IFNAR^−/−^ Mice

Each group of three IFNAR^−/−^ mice was anesthetized with midazolam, medetomidine, and a butorphanol tartrate combination, was intraperitoneally inoculated with 1 × 10^7^ TCID_50_ of HRTV at a volume of 200 μL per mouse, and was treated with antiviral agents, as described below. For the mock infection, the same volume of DMEM-2FBS was used. The favipiravir-treated and ribavirin-treated mice were orally administered 60 mg/kg of favipiravir (FUJIFILM Toyama Chemical Co., Ltd., Tokyo, Japan) and 100 mg/kg of ribavirin (FUJIFILM Wako Chemicals), respectively, dissolved in a 0.5% methyl cellulose (Sigma-Aldrich, St. Louis, MO, USA) solution, at a volume of 200 μL per mouse, once a day for 5 days. The placebo-treatment mice were administered the same volume of 0.5% methylcellulose once a day for 5 days. The first administration of each compound was initiated immediately after the HRTV inoculation. Each mouse was monitored daily for the development of clinical signs, including bodyweight, and blood samples were obtained by tail-vein puncture at 2 and 4 days postinfection to measure the viral RNA levels.

### 2.5. Measurement of Viral RNA Levels in Blood

Total RNA was extracted from 20 μL of blood using the High Pure Viral Nucleic Acid kit (Roche Applied Science, Penzberg, Germany). The HRTV NS gene levels were calculated using a QuantiTect Probe RT-PCR kit (Qiagen, Hilden, Germany), as described previously [29]. Briefly, the forward primer (5′- TGCAGGCTGCTCATTTATTC -3′), reverse primer (5′- CCTGTGGAAGAAACCTCTCC -3′), and probe (5′- FAM- CCTGACCTGTCTCGACTGCCCA -BHQ-1 -3′) were used for the RT-PCR. Fluorescent-signal levels were detected using an Applied Biosystems 7500 system (Applied Biosystems, Waltham, MA, USA).

Control RNA, which included the HRTV NS target gene, was synthesized as described below. The cDNA fragment containing the ORF of the HRTV NSs was cloned into the EcoRI site of pBluescript II KS to construct pBS-HRTV-NSs. The RNA was synthesized from HindIII-digested pBS-HRTV-NSs using a mMESSAGE mMACHINE T7 Transcription Kit (Invitrogen, Waltham, MA, USA), according to the manufacturer’s protocol.

## 3. Results

### 3.1. Susceptibility of IFNAR^−/−^ Mice to HRTV

**Figure 1:**
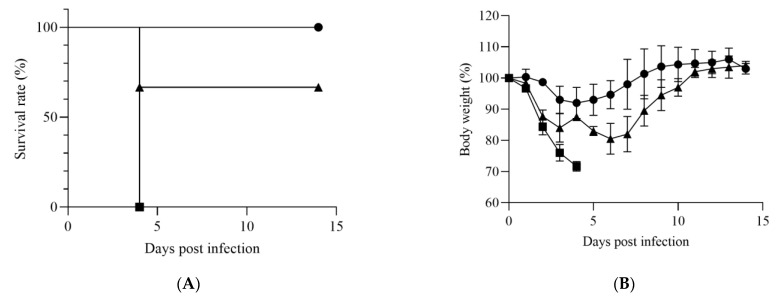

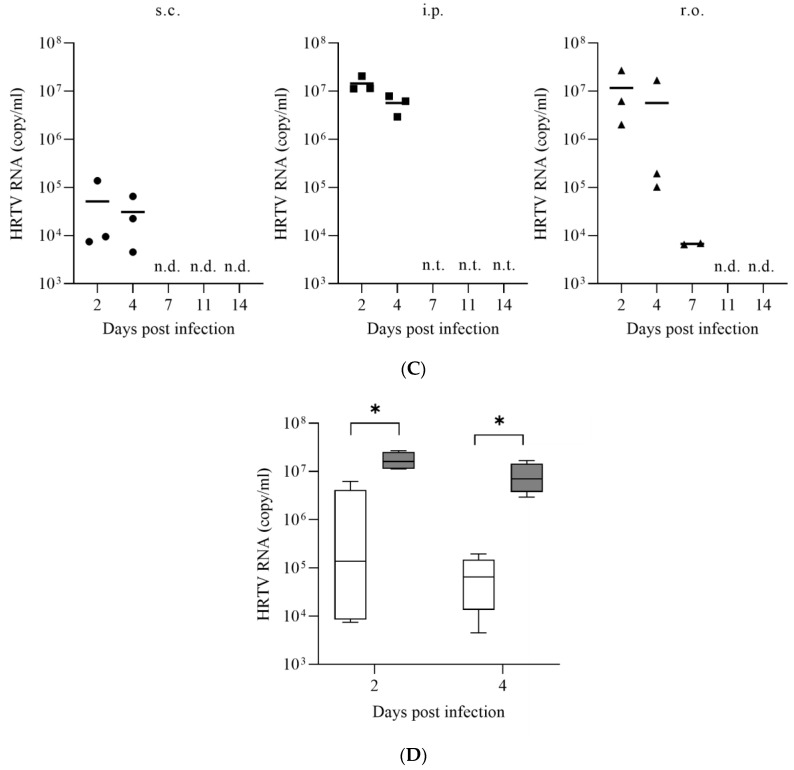
IFNAR^−/−^ mice were infected with 1 × 10^7^ TCID_50_ of HRTV subcutaneously (closed circles), intraperitoneally (closed squares), or retro-orbitally (closed triangles). (**A**) Survival rate of the infected IFNAR^−/−^ mice (*n* = 3). (**B**) Sequential changes in bodyweights of IFNAR^−/−^ mice (*n* = 3). Relative weights were calculated and are shown as means with standard deviations. (**C**) The copy numbers of HRTV RNA in blood samples collected from the infected IFNAR^−/−^ mice on days 2, 4, 7, 11, and 14 postinfection (*n* = 3). n.d.: not determined. n.t.: not tested. (**D**) Comparison of levels of HRTV RNA between IFNAR^−/−^ mice that survived and those that died of HRTV infection. The white box and gray box indicate the HRTV RNA levels of the mice that survived and those that reached the endpoint, respectively. Significance was determined by Mann–Whitney test. * *p* < 0.05.

The susceptibility of adult IFNAR^−/−^ mice to HRTV infection was investigated. IFNAR^−/−^ mice were inoculated with HRTV at a dose of 1 × 10^7^ TCID_50_, which is the maximum dose of HRTV that can be infected following the guidelines approved by the Animal Care and Use Committee of the NIID, through either the subcutaneous, intraperitoneal, or retro-orbital routes. All mice infected subcutaneously showed weight loss, but survived (Figure 1A,B). The mean copy numbers of the HRTV RNA in the blood of the subcutaneously infected mice were 5.2 × 10^4^ copies/mL and 3.1 × 10^4^ copies/mL at days 2 and 4 postinfection, respectively, and they became below detectable levels at days 7, 11, and 14 postinfection (Figure 1C, left). The mice infected intraperitoneally showed severe clinical signs, such as ruffled fur, hunched postures, and acute weight loss. Furthermore, all intraperitoneally infected mice reached the endpoint within 4 days postinfection (Figure 1A,B). The mean copy numbers of the HRTV RNA in the blood of the intraperitoneally infected mice were 1.4 × 10^7^ copies/mL and 5.7 × 10^6^ copies/mL at 2 and 4 days postinfection, respectively (Figure 1C, center). The retro-orbitally infected mice showed intermediate symptoms in terms of severity between the subcutaneously infected mice and the intraperitoneally infected mice, and two of the three mice survived (Figure 1A). All three mice showed weight loss; however, the weight recovered from 7 days postinfection in the surviving retro-orbitally infected mice (Figure 1B). The mean copy numbers of the HRTV RNA in the blood of the retro-orbitally infected mice were 1.2 × 10^7^, 5.7 × 10^6^, and 6.7 × 10^3^ copies/mL at 2, 4, and 7 days postinfection, respectively, and they were below detectable levels at 11 and 14 days postinfection (Figure 1C). The levels of HRTV RNA in the blood were significantly higher in the mice that reached the endpoint than in those that survived both 2 and 4 days postinfection, with statistical significance (Figure 1D).

**Figure 2:**
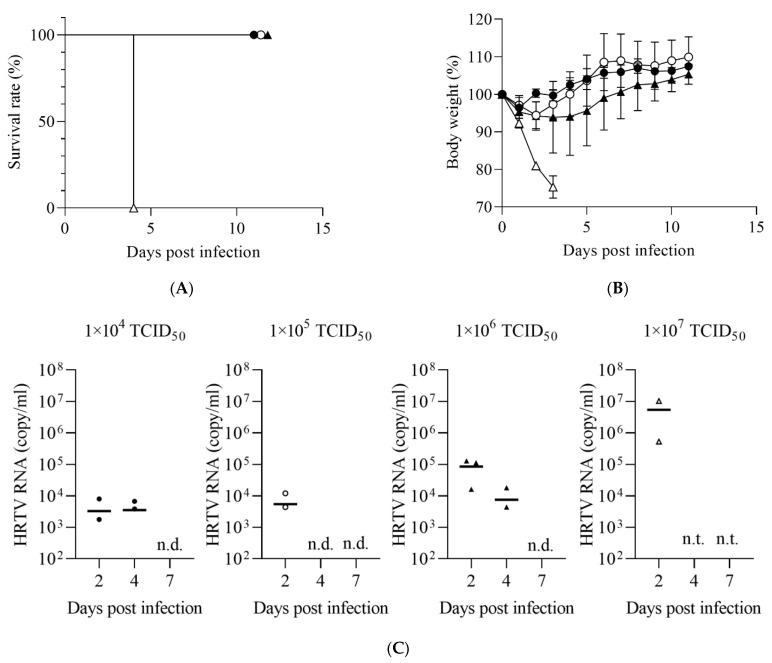
IFNAR^−/−^ mice were infected intraperitoneally with 1 × 10^4^, 1 × 10^5^, 1 × 10^6^, and 1 × 10^7^ TCID_50_ of HRTV, which are indicated by the closed circles, open circles, closed triangles, and open triangles, respectively. Each group consisted of three mice, except for the 1 × 10^7^ TCID_50_-HRTV-infected mice group, in which two mice were used. (**A**) Survival rate of infected IFNAR^−/−^ mice. (**B**) Sequential body weight change of each group of IFNAR^−/−^ mice. Relative weights were calculated and are shown as means with standard deviations. (**C**) The copy numbers of HRTV RNA in blood samples collected from infected IFNAR^−/−^ mice on days 2, 4, and 7 postinfection. n.d.: not determined. n.t.: not tested.

To determine the 50% lethal dose (LD_50_) of HRTV in IFNAR^−/−^ mice when infected intraperitoneally, the IFNAR^−/−^ mice were inoculated intraperitoneally with HRTV at doses of 1 × 10^4^, 1 × 10^5^, 1 × 10^6^, or 1 × 10^7^ TCID_50_. The survival rate, sequential change in weight, and clinical signs were observed for 11 days postinoculation. IFNAR^−/−^ mice infected with 1 × 10^7^ TCID_50_ of HRTV showed severe weight loss, ruffled fur, and hunched postures, and they died within 4 days postinfection (Figure 2A,B). In contrast, IFNAR^−/−^ mice infected with 1 × 10^4^, 1 × 10^5^, and 1 × 10^6^ TCID_50_ of HRTV showed no obvious clinical signs, except for slight weight loss, and they survived (Figure 2A,B). The LD_50_ of HRTV to IFNAR^−/−^ mice when infected intraperitoneally was calculated to be 3.2 × 10^6^ TCID_50_. The mean copy numbers of the HRTV RNA in the blood of IFNAR^−/−^ mice infected with 1 × 10^4^, 1 × 10^5^, 1 × 10^6^, and 1 × 10^7^ TCID_50_ of HRTV at 2 days postinfection were 3.4 × 10^3^, 5.7 × 10^3^, 8.6 × 10^4^, and 5.4 × 10^6^ copies/mL, respectively (Figure 2C). The mean copy numbers of the HRTV RNA in the blood of IFNAR^−/−^ mice infected with 1 × 10^4^, 1 × 10^5^, and 1 × 10^6^ TCID_50_ of HRTV at 4 days postinfection were 3.7 × 10^3^, < 0.5 × 10^3^ (under detectable level), and 7.7 × 10^3^ copies/mL, respectively (Figure 2C). All IFNAR^−/−^ mice infected with 1 × 10^7^ TCID_50_ of HRTV died by 4 days postinfection (Figure 2A). The copy numbers of the HRTV RNA in the blood of all surviving mice were below detectable levels at 7 days postinfection or later (Figure 2C).

### 3.2. Antiviral Activities of Favipiravir and Ribavirin against HRTV In Vivo

**Figure 3:**
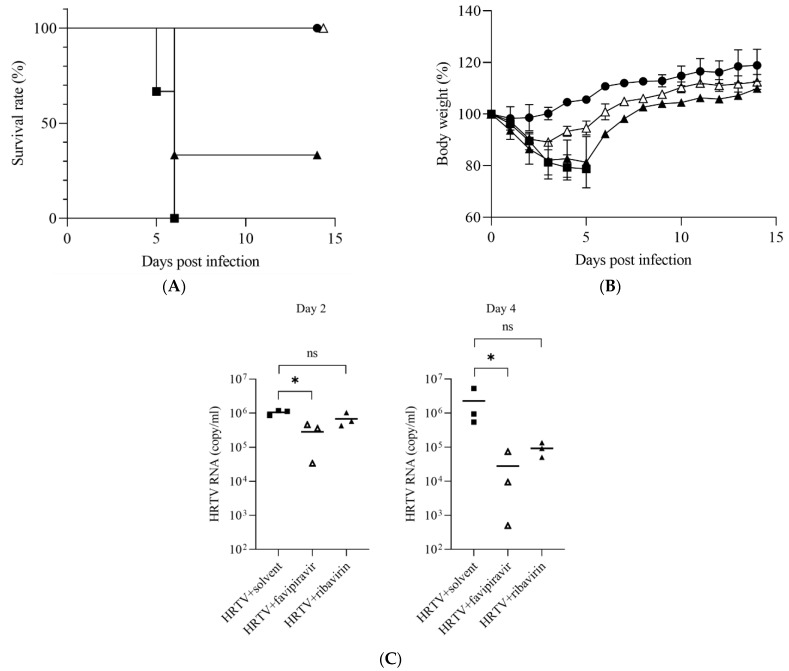
The in vivo efficacy of favipiravir and ribavirin in the treatment of IFNAR^−/−^ mice infected with HRTV. IFNAR^−/−^ mice were mock infected, indicated by the closed circles, or inoculated with 1 × 10^7^ TCID_50_ of HRTV intraperitoneally and treated with 60 mg/kg/day of favipiravir, 100 mg/kg/day of ribavirin, or the same amount of 0.5% methylcellulose, indicated by the open triangle, closed triangle, or closed square, respectively, from 0 to 4 days postinfection. (**A**) The survival rates of the mice (*n* = 3). (**B**) Bodyweights of the IFNAR^−/−^ mice. The relative bodyweights are shown as means with standard deviations (*n* = 3). (**C**) The copy numbers of HRTV RNA in blood of the infected mice at 2 and 4 days postinfection. The levels of significance were determined in comparison with the results of the group of mice infected with HRTV and treated with solvent using a Kruskal–Wallis test. * *p* < 0.05.

The in vivo activities of favipiravir and ribavirin against HRTV infection in IFNAR^−/−^ mice were evaluated. The IFNAR^−/−^ mice were intraperitoneally inoculated with 1 × 10^7^ TCID_50_ of HRTV and were then administered 60 mg/kg/day of favipiravir, 100 mg/kg/day of ribavirin, or the same amount of 0.5% methylcellulose solvent for 5 consecutive days from 0 to 4 days postinfection, taking the day that the mice were infected with HRTV as day 0. All mice treated with the solvent showed acute weight loss and died within 6 days of HRTV infection (Figure 3A,B). Thirty-three percent of the mice treated with ribavirin survived HRTV infection, while all the mice treated with favipiravir survived (Figure 3A). The mice treated with ribavirin and those treated with favipiravir showed significant weight loss after HRTV infection compared with the mock-infected mice. However, the bodyweights of the surviving mice recovered from days 4 and 6 postinfection when treated with favipiravir and ribavirin, respectively (Figure 3B). The mean copy numbers of the HRTV RNA in the solvent-, favipiravir-, and ribavirin-treated mice were 1.1 × 10^6^, 2.9 × 10^5^, and 6.8 × 10^5^ copies/mL at 2 days postinfection, respectively (Figure 3C, left). The copy numbers of the HRTV RNA of the favipiravir-treated mice were significantly lower than those of the solvent-treated mice at 2 days postinfection, whereas the copy numbers of the HRTV RNA of the ribavirin-treated mice were the same as those of the solvent-treated mice (Figure 3C, left). The mean copy numbers of the HRTV RNA in the solvent-, favipiravir-, and ribavirin-treated mice at 4 days postinfection were 2.3 × 10^6^, 4.2 × 10^4^, and 9.3 × 10^4^ copies/mL, respectively (Figure 3C, right). The difference in the copy numbers of the HRTV RNA between the solvent- and favipiravir-treated-mice observed at 2 days postinfection was less than 1 log but was significant; however, the difference became more apparent at 4 days postinfection (Figure 3C).

## 4. Discussion

In this study, the adult IFNAR^−/−^ mice intraperitoneally infected with equal to or less than 1 × 10^6^ TCID_50_ of HRTV showed no clinical signs, except for slight weight loss (Figure 2). The results obtained in the present study were consistent with those of a previous study, in which it was reported that weaning IFNAR^−/−^ mice showed weight loss compared with mock-infected mice, but did not die when the mice were infected with HRTV intraperitoneally at a dose of a 2.5 × 10^5^ 50% cell-culture-infective dose (CCID_50_) [20]. In contrast, all intraperitoneally inoculated adult IFNAR^−/−^ mice with 1 × 10^7^ TCID_50_ of HRTV died (Figure 2A). These results suggested that the severity of HRTV infection was dose dependent.

The clinical signs and outcomes of IFNAR^−/−^ mice infected with 1 × 10^7^ TCID_50_ of HRTV intraperitoneally were more severe than those of IFNAR^−/−^ mice infected with 1 × 10^7^ TCID_50_ of HRTV subcutaneously or retro-orbitally (Figure 1). The precise mechanism of the difference in the severity among the three groups that differed by the HRTV inoculation route was not revealed in the present study. However, the HRTV RNA levels in the blood of the surviving mice at days 2 and 4 postinfection were significantly lower than those of the mice that reached the endpoint (Figure 1D). The virus inoculation routes may influence the severity because of the accessibility of HRTV to target cells, which has not yet been determined, and the way of the induction of the host immune response depends on the route of virus inoculation [30].

The IFN signaling pathway played a critical role in preventing the hosts from having HRTV infection [20,27,31]. In this study, IFNAR^−/−^ mice showed severe illness when infected with 1 × 10^7^ TCID_50_ of HRTV intraperitoneally. The LD_50_ of intraperitoneal HRTV infection in IFNAR^−/−^ mice was 3.2 × 10^6^ TCID_50_ in the present study, while immunocompetent C57BL/6 mice did not show any symptoms of infection with HRTV [27]. This indicates the importance of type I IFN signaling in the pathogenesis of HRTV infection in mice. In addition, the LD_50_ of HRTV in AG129 mice, which were deficient in both type I and type II IFN signaling, intraperitoneally infected, were nine plaque-forming units (PFUs). This was much lower than that of the C57BL/6J mice-based IFNAR^−/−^ mice. This evidence supports previous studies, which showed the role of type II IFN signaling in the pathogenesis of HRTV infection [20,27].

The development of this HRTV-infection animal model enabled us to evaluate the in vivo efficacy of antiviral agents, such as favipiravir and ribavirin. The efficacy of favipiravir and ribavirin against HRTV infection has already been demonstrated using the signal transducer and activator of transcription 2 (STAT2) knock-out (KO) hamsters, showing that favipiravir, but not ribavirin, was effective against HRTV infection in vivo [20]. However, the efficacy was shown only by comparing the levels of weight change between the groups treated with each antiviral agent and placebo [20]. In contrast, the efficacy of antiviral agents against HRTV infection in vivo was evaluated based not only on the levels of weight loss, but also on other clinical signs, including the survival rate and levels of HRTV RNA in the blood. The favipiravir treatment significantly lowered the weight loss and HRTV RNA levels in the blood, and the survival rate recovered to 100% (Figure 3). This suggests that favipiravir may be effective in the treatment of animals with acute lethal HRTV infection. In contrast, the ribavirin treatment did not result in significant changes in the weight loss and HRTV RNA levels in the blood compared with the solvent-treated group (Figure 3B,C). This suggests that ribavirin has little, if any, effect on HRTV infection.

There are several advantages of the HRTV-infection model that uses IFNAR^−/−^ mice compared with previously reported models [20,27]. First, although the conclusions of the studies on the efficacy of antiviral agents against HRTV infection using STAT2 KO hamsters [20] and those of the present study, in which IFNAR^−/−^ mice were used, were the same, the efficacies were more clearly shown in the IFNAR^−/−^ mouse model. The clinical signs shown in IFNAR^−/−^ mice were more severe than those in STAT2 KO hamsters [20], and the efficacy could be evaluated by measuring the levels of HRTV RNA in the blood. This result indicates that the IFNAR^−/−^ mouse model is more sensitive to HRTV infection than the STAT2 KO hamster model in terms of disease progression, which makes it convenient to evaluate the efficacy of antiviral agents against HRTV infection. In addition, there are drawbacks to using hamsters as an animal model, as there are limitations to the availability of reagents for studying the immune responses in hamsters compared with mice [32]. Second, although AG129 mice are highly susceptible to HRTV infection, histopathological lesions are limited to spleen, liver, and renal, in which neutrophilic infiltration and an increased number of mononuclear cells with numerous viral antigen-positive cells have been observed [27,28]. HRTV can cause widely disseminated infection with severe shock and multisystem organ failure, including necrosis of the liver, gastrointestinal symptoms, renal failure, splenic infarction, and hemorrhage in human patients [12,16]. Both type I and II IFN signaling are critical during virus infection to not only limit the virus replication and initiate an appropriate antiviral immune response, but also to negatively regulate this response to minimize tissue damage [33]. Viral infections sometimes cause hyper immune responses, which have adverse effects on organs and lead to high pathogenicity and mortality [34,35]. Hyper immune responses have been also reported in SFTS patients [36]. IFNAR^−/−^ mice lack only type I IFN, and they reflect a more normal immune response in the host compared with AG129 mice, which cause the pathogenesis of HRTV infection. Thus, this animal model might be useful for understanding the mechanism of the pathogenesis of the acute phase of HRTV infection, and for evaluating the efficacy of antiviral reagents and vaccines against acute HRTV infection. We are now performing experiments to reveal the precise pathogenesis of HRTV infection in IFNAR^−/−^ mice. As clinical reports on HRTV disease are limited [11,12,16,37,38], pathological examinations and clinical research on HRTV patients are also important to understand the mechanism of the pathogenesis of HRTV infection. In addition, as IFNAR^−/−^ mice are generally used as animal models of SFTSV infection [21,23,25,26], it might be useful to compare the viral replication and mechanism of the pathogenesis between SFTSV and HRTV in vivo using IFNAR^−/−^ mice.

In conclusion, a novel lethal HRTV-infection model was established using IFNAR^−/−^ mice. This animal model was shown to be useful for evaluating the efficacy of antiviral agents and possible vaccines.

## Data Availability

Not applicable.
